# Disruption of the autism-associated gene *SCN2A* alters synaptic development and neuronal signaling in patient iPSC-glutamatergic neurons

**DOI:** 10.3389/fncel.2023.1239069

**Published:** 2024-01-16

**Authors:** Chad O. Brown, Jarryll A. Uy, Nadeem Murtaza, Elyse Rosa, Alexandria Alfonso, Biren M. Dave, Savannah Kilpatrick, Annie A. Cheng, Sean H. White, Stephen W. Scherer, Karun K. Singh

**Affiliations:** ^1^Department of Biochemistry and Biomedical Sciences, Faculty of Health Sciences, McMaster University, Hamilton, ON, Canada; ^2^Krembil Research Institute, University Health Network, Toronto, ON, Canada; ^3^Department of Laboratory Medicine and Pathobiology, Faculty of Medicine, University of Toronto, Toronto, ON, Canada; ^4^Department of Molecular Genetics, Faculty of Medicine, University of Toronto, Toronto, ON, Canada; ^5^SickKids Research Institute, The Hospital for Sick Children, Toronto, ON, Canada

**Keywords:** sodium channel, autism, human neuron, synapse, dendrite

## Abstract

*SCN2A* is an autism spectrum disorder (ASD) risk gene and encodes a voltage-gated sodium channel. However, the impact of ASD-associated SCN2A *de novo* variants on human neuron development is unknown. We studied SCN2A using isogenic *SCN2A*^–/–^ induced pluripotent stem cells (iPSCs), and patient-derived iPSCs harboring a *de novo* R607* truncating variant. We used Neurogenin2 to generate excitatory (glutamatergic) neurons and found that *SCN2A*^+/*R*607*^ and *SCN2A*^–/–^ neurons displayed a reduction in synapse formation and excitatory synaptic activity. We found differential impact on actional potential dynamics and neuronal excitability that reveals a loss-of-function effect of the R607* variant. Our study reveals that a *de novo* truncating *SCN2A* variant impairs the development of human neuronal function.

## Introduction

Autism spectrum disorder (ASD) is a childhood-onset, heterogeneous group of neurodevelopmental disorders that can have a range of severities between individuals. ASD is characterized by deficits in social communication, restricted and repetitive patterns of behavior, or interests (Ofner et al., 2018). Large-scale genetic sequencing studies have highlighted the contributions of genetic variants in hundreds of genes to the underlying etiology, including rare-inherited and *de novo* variants being a major contributor of risk (Iossifov et al., 2014; Sanders et al., 2015; Yuen et al., 2017; Grove et al., 2019; Ruzzo et al., 2019; Satterstrom et al., 2020). Whole exome and genome sequencing studies of ASD cohorts have identified multiple *de novo* genetic variants within SCN2A, which has led to SCN2A being one of the strongest individual candidate risk genes for ASD (Sanders et al., 2012; Wang et al., 2016; Satterstrom et al., 2019, 2020; Crawford et al., 2021). SCN2A encodes the neuronal α-subunit of the voltage-gated sodium channel Na_*V*_1.2, which is predominately expressed in excitatory glutamatergic neurons within the cortex in the axon initial segment where it regulates action potential generation (Hu et al., 2009; Bender and Trussell, 2012; Kole and Stuart, 2012; Spratt et al., 2019).

Understanding how variants in *SCN2A* contribute to disease is important as this may inform potential therapies or long-term clinical outcomes. Cell line and computational studies have been instrumental in understanding the potential impact of the *SCN2A* variants (Ben-Shalom et al., 2017; Begemann et al., 2019; Que et al., 2021; Echevarria-Cooper et al., 2022). These studies have indicated that *SCN2A* gain-of-function variants lead to increased channel function and epileptic encephalopathy. Loss-of-function (LoF) variants in *SCN2A* are associated with reduced channel function, leading to ASD and intellectual disability (Ben-Shalom et al., 2017; Sanders et al., 2018; Begemann et al., 2019). Mice heterozygous for loss of *Scn2a* have defects in spatial memory and learning, and social behavior (Ogiwara et al., 2018; Spratt et al., 2019; Tatsukawa et al., 2019). Additionally, Na_*v*_1.2 channels in deep layer (5/6) prefrontal cortical excitatory neurons mediate backpropagation of action potentials to dendrites, postsynaptic calcium influx, synaptic function and plasticity (Spratt et al., 2019, 2021). These studies suggest heterozygous loss of Scn2a function results in abnormal excitatory circuit development in the cortex. While mouse models provide important insights, there are very few human-derived models to explore disease phenotypes (Mis et al., 2019). We have previously showed that isogenic *SCN2A*^–/–^ glutamatergic neurons display reduced synaptic activity (Deneault et al., 2018). Currently, *SCN2A* has not been modeled using an ASD individual-specific neuronal system.

To date, little is known about how ASD patient-specific protein truncating mutations in *SCN2A* affect neuron function and development. Therefore, we generated induced pluripotent stem cells (iPSCs) from an ASD individual harboring an *SCN2A*^+/*R*607*^ variant and their sex-matched parental control, recruited from the Autism Speaks MSSNG project.^1^ Further, there is incomplete penetrance of seizure phenotypes in *SCN2A* patients and that some heterozygous models may not recapitulate clinical observations (Ogiwara et al., 2018; Sanders et al., 2018; Fallah and Eubanks, 2020). In such cases, it would be of benefit to use a homozygous knockout model to exacerbate and identify possible cellular mechanisms that are disrupted. We therefore, generated a new isogenic *SCN2A*^–/–^ iPSC line to identify functional similarities in neuronal phenotypes between complete and partial loss of SCN2A protein.

**FIGURE 1:**
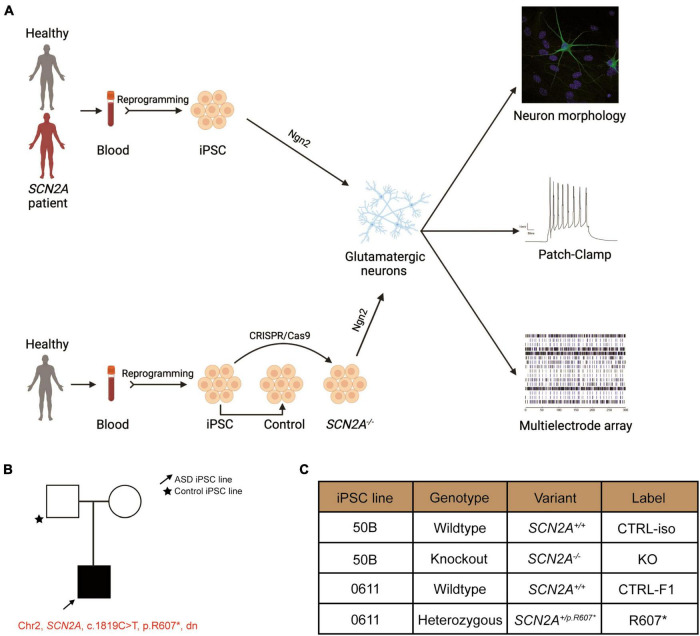
Experimental pipeline to probe cellular and molecular consequences of SCN2A deficiency in iPSC-derived iNeurons. See also Supplementary Figure 1. **(A)** Experimental pipeline to test cellular and signaling function. **(B)** *SCN2A* patient and family pedigree. **(C)** Classification of iPSC lines used in this study and their associated naming convention, genotype and genetic presentation of *SCN2A*.

Here, we used the Neurogenin2 (NGN2) expression protocol (Zhang et al., 2013) to generate induced glutamatergic neurons (iNeurons) for electrophysiological studies (Figure 1A). We found that isogenic *SCN2A*^–/–^ iNeurons produce paradoxical hyperexcitable phenotypes in iNeurons, similar to rodent *Scn2a* KO neurons (Spratt et al., 2019, 2021; Zhang et al., 2021). Further electrophysiological analysis of isogenic *SCN2A*^–/–^ and *SCN2A*^+/ R 607*^ iNeurons revealed a similar reduction in synapse development, synaptic transmission and neuronal network activity. Our results provide an in-depth characterization of the ASD-associated R607* variant in *SCN2A* through impaired neuron development through disruptions in synaptic function and morphology and neuronal network activity.

## Materials and methods

### Approval and generation of iPSCs

All pluripotent stem cell work was approved by the Canadian Institutes of Health Research Stem Cell Oversight Committee. Blood was taken from individuals with the approval from SickKids Research Ethics Board after informed consent was obtained, REB approval file 1000050639. This study was also approved by the Hamilton Integrated Research Ethics Board, REB approval file #2707. CD34+ blood cells were verified using flow cytometry and collected for iPSC reprogramming. All iPSCs were generated by Sendai virus reprogramming and clonal expansion using the CytoTune—iPSC 2.0 kit (ThermoFisher) to deliver the reprogramming factors. Once colonies were large enough (approximately 15–17 days post Sendai transduction), each colony was transferred to 1 well of a 12-well plate coated with irradiated mouse embryonic fibroblasts (MEFs) and plated in iPSC media [DMEM/F12 supplemented with 10% KO serum, 1x non-essential amino acids, 1x GlutaMAX, 1 mM β-mercaptoethanol, and 16 ng/mL basic fibroblast growth factor (bFGF)]. Once iPSCs were expanded and established they were transitioned to matrigel coated plates and grown in mTeSR1 (STEMCELL Technologies) and subsequent passaging continued to use ReLeSR (STEMCELL Technologies). iPSC lines without karyotypic abnormalities were used and this was verified by G-band karyotyping performed by the Centre for Applied Genomics (The Hospital for Sick Children). To verify the expression of pluripotent markers OCT4 and NANOG, immunocytochemistry was performed. iPSC lines were validated to be mycoplasma free using the MycoAlert detection kit (Lonza).

### Generation of *SCN2A* KO cells

A 3x stop premature termination codon (StopTag) was designed similarly to our previously described method for introducing a StopTag into the DNA to knock out the expression of genes of interest (Deneault et al., 2018). This 3x StopTag was delivered by a synthesized single-stranded oligodeoxynucleotide (ssODN) containing two 50-nucleotide-long homology arms with the StopTag, V5 epitope and *Eco*RI restriction site sequences coding 59 nucleotides. The knock in site was selected to situate the StopTag 80 bp upstream of the exon 5–6 junction, to increase the likelihood of transcript knockout by the nonsense-mediated decay pathway (Neu-Yilik et al., 2011). An iPSC line derived from a health neurotypical control named “50B” previously described was used to insert the StopTag and generate an isogenic *SCN2A* KO line (Deneault et al., 2018). The StopTag ssODN template, a pSpCas9(BB)-2A-GFP plasmid (Addgene, catalog no. 48138) and paired gRNAs targeting exon 5 of *SCN2A* were nucleofected into the 50B iPSCs using the Amaxa 4D-nucleofector with code CA137. GFP expressed cells were isolated 48 h after nucleofection and clonally grown. Droplet digital PCR and dilution culture steps previously described were used to enrich for *SCN2A* KO populations (Miyaoka et al., 2014; Deneault et al., 2018). The purified *SCN2A* KO wells were expanded and assayed for pluripotency, karyotypic abnormalities, mycoplasma (Lonza MycoAlert) and sequencing validation of StopTag insertion (Supplementary Figure 1).

### Induction of iPSCs into glutamatergic neurons

We sought to explore functional differences of the 2 genetics models of *SCN2A* deficiency. For this, we differentiated the newly generated iPSCs into excitatory glutamatergic neurons. Since previous findings showed inhibitory neurons were unaffected by *SCN2A* deficiency, we required an established system to explore excitatory neuron driven differences (Ogiwara et al., 2018; Spratt et al., 2019; Wang et al., 2021). In order to rapidly upscale experiments and focus on excitatory neurons, we used the previously published constitutive expression protocol of NGN2 to generate homogeneous populations of glutamatergic neurons (Zhang et al., 2013). These iNeurons displayed stable membrane, firing and synaptic properties when co-cultured with mouse glial cells by DIV 21 (Zhang et al., 2013, 2018). Importantly as we previously described, this protocol provided consistent differentiation levels between cell lines derived from different participants (Deneault et al., 2018, 2019). We modified this protocol by inducing NGN2 for 3 days starting at DIV 1 and puromycin selecting for 2 days starting at DIV 2 and adding mouse glial cells at DIV 5. Half-iNeuron media (iNI) (Neurobasal media, 1x SM1, 1x GlutaMAX 1x pen/strep, 1 μg/mL laminin, 10 ng/uL BDNF and 10 ng/uL GDNF) changes were performed every other day. Patch-clamp recordings were generated between DIV 24 and 27 post-NGN2-induction.

### Droplet digital PCR

RNA from iNeurons cultured were extracted using the RNeasy Mini kit (Qiagen). cDNA was generated using the qScript cDNA SuperMix (QuantaBio). ddPCR was performed as previously described (Deneault et al., 2018). Quantification was normalized to the control of each condition and run along a no template control. The data was analyzed and produced on the QuantaSoft software (Bio-Rad). The following assays were used: *SCN2A* exon 4–5 (Hs01109883_m1, ThermoFisher) and TBP (Hs00427620_m1, ThermoFisher).

### PCR validation of R607* patient iPSC

Genomic DNA was extracted from CTRL-F1 and R607* iPSCs using the Qiagen DNeasy Blood & Tissue kit following the manufacturer’s protocol. A region containing the single nucleotide variant was cloned out by PCR using the following primers: FOR (TTTTCAGCTTCAGAGGTC and REV AGACCACACCATTGCAGTCCAC). For quantifying and validating the heterozygosity of the R607* patient line, the prior PCR product was cloned into sequencing plasmids using the CloneJET PCR Cloning Kit (Thermo Fisher Scientific) following the manufacturer’s protocol. Colonies were picked from carbenicillin-treated LB agar plates, and plasmids were purified and sent for sanger sequencing (The Centre for Applied Genomics) using the CloneJET Forward primer.

### Proteomic profiling by tandem-mass-tag-based mass spectrometry

Approximately 150 μg of total protein was extracted from 3 independent NGN-2 transductions cultured without mouse glial cells at DIV14 of each familial control and proband patient line (total *n* = 3 per cell line) using 8 M urea and 100 mM ammonium bicarbonate. These protein samples were reduced with 10 mM tris(2-carboxyethyl)phosphine for 45 min at 37°C, alkylated with 20 mM iodoacetamide for 45 min at room temperature, and digested by trypsin (Promega) [1:50 (w/w) enzyme-to- protein ratio] overnight at 37°C. The resulting peptides were desalted with the 10 mg SOLA C18 Plates (Thermo Scientific), dried, labeled with 16-plex tandem mass tag reagents (Thermo Scientific) in 100 mM triethylammonium bicarbonate, and quenched with 5% hydroxylamine before being pooled together. A total of 40 μg of the pooled sample was separated into 36 fractions (2 min each) by high-pH reverse- phase liquid chromatography (RPLC) using a homemade C18 column (200 μm × 30 cm bed volume, Waters BEH 130 5 μm resin) running a 70 min gradient from 11 to 32% acetonitrile- 20 mM ammonium formate (pH 10) at flow rate of 5 μL/min. Each fraction was then loaded onto a homemade trap column (200 μm × 5 cm bed volume) packed with POROS 10R2 10 μm resin (Applied Biosystems), followed by a homemade analytical column (50 μm × 50 cm bed volume) packed with Reprosil-Pur 120 C18-AQ 5 μm particles with an integrated Picofrit nanospray emitter (New Objective). LC-MS experiments were performed on a Thermo Fisher Ultimate 3000 RSLCNano UPLC system that ran a 3 h gradient (11-38% acetonitrile-0.1% formic acid) at 70 nL/min coupled to a Thermo QExactive HF quadrupole-Orbitrap mass spectrometer. A parent ion scan was performed using a resolving power of 120 000; then, up to 30 of the most intense peaks were selected for MS/MS (minimum ion counts of 1,000 for activation) using higher energy collision-induced dissociation (HCD) fragmentation. Dynamic exclusion was activated such that MS/MS of the same m/z (within a range of 10 ppm; exclusion list size = 500) detected twice within 5 s was excluded from the analysis for 30 s.

### Proteomic data analysis

LC-MS data were searched against a UniProt human protein database (ver 2017-06, 25020 entries) for protein identification and quantification by Protein Discover software (Thermo). Protein quantification was normalized by taking the sum of each TMT channel and normalizing it to a control sample per family. Protein quantities were log2 transformed to calculate fold changes and *P*-values were calculated using Student’s *t*-test.

### Immunocytochemistry

iPSCs were washed gently 2 times with PBS and fixed in 4% paraformaldehyde in PBS for 8 min at room temperature. The cells were then washed 2 times with PBS and left overnight in 4°C. The next day, the cells were permeabilized in −30°C with ice cold methanol for 10 min. The cells were then washed 2 times with PBS for 8 min and incubated primary antibodies overnight at 4°C. The following day, the cells were washed 3 times with PBS for 8 min. Secondary antibodies were incubated for 1 h at room temperature covered with aluminum foil, followed by 3 washes in PBS for 8 min. After the washes, 300 mM DAPI in PBS was incubated for 8 min, followed by 2 washes with PBS. Coverslips were then quickly dried with a Kimwipe, and mounted on VistaVision glass microscope slides (VWR) with 10 μL of Prolong Gold Anti-Fade mounting medium (Life Technologies). Mounted coverslips were allowed to cure overnight in a dark slide box at room temperature. Images were acquired using a Zeiss LSM700 confocal microscope.

On DIV 25, iNeurons were fixed at room temperature in 4% paraformaldehyde in PBS for 15 min. The cells were then washed 3 times for 10 min with PBS, then blocked and permeabilized (B/P) with a B/P solution containing (0.3% Triton-X, 10% Donkey Serum, and PBS) for 1 h. The cells were then incubated overnight at 4°C with primary antibodies in B/P solution. The next day, cells were washed 3 times for 10 min in PBS and incubated with secondary antibodies in B/P solution for 1.5 h at room temperature and covered with aluminum foil. The cells were then washed 3 times for 10 min and incubated with 300 mM DAPI for 8 min. The cells were then washed 1 time with PBS for 10 min. Coverslips were then quicky dried with a Kimwipe, and mounted on VistaVision glass microscope slides (VWR) with 10 μL of Prolong Gold Anti-Fade mounting medium (Life Technologies). Mounted coverslips were allowed to cure overnight in a dark slide box at room temperature. Images were acquired using a Zeiss LSM700 confocal microscope.

Synaptic morphology was processed and analyzed with ImageJ software. The Synapsin1 antibody was co-immunostained with MAP2 to determine dendrites with presynaptic puncta. Three biological replicates were used for each line with the data generated from five iNeurons per replicate per condition. A total of 15 iNeurons per condition per line were used with two dendrites of equal dimensions used per iNeuron. Data represent the number of synaptic puncta averaged by two dendrites per iNeuron within 30 μm segments. The same images were used to calculate dendrite complexity. This was determined by counting the number of MAP2-positive primary dendrites branching from the soma.

### Multi-electrode array

All recordings were performed using 48-well clear bottom MEA plates (Axion Biosystems), consisting of 16 electrodes per well. Plates were coated with filter-sterilized 0.1% polyethyleneimine solution in borate buffer pH 8.4 for 2 h at 37°C, washed with water four times and dried overnight. 40,000 DIV 4 doxycycline iNeurons were seeded in a 20 uL drop of iNI media at the center of each well for 1.5 h, then an additional 150 uL of iNI media was added. The day after, 20,000 mouse astrocytes per well were seeded on top of iNeurons in 150 uL per well of iNI media. Mouse astrocytes were prepared from postnatal day 1 CD-1 mice as described (Kim and Magrané, 2011). Half media changes were performed every other day with iNI media until the endpoint of experiments. The electrical activity of neurons was measured a minimum of once-a-week post-seeding onto MEA plates using the Axion Maestro MEA reader (Axion Biosystems). On the day of recording, MEA plates were equilibrated for 5 min on the pre-warmed reader at 37°C. Real-time spontaneous neural activity was recorded for 10 min to use for offline processing. Recordings were sampled at 10 kHz, and filtered with a bandpass filter from 200 Hz to 3 kHz. A threshold of greater than 6 standard deviations was used to detect spikes and separate noise. Electrodes were considered active if a minimum of 5 spikes were detected per minute. Wells that were unable to generate 10 active electrodes of the 16 by DIV 42 were not used for analysis. Bursts were defined as a minimum of 5 spikes with a maximum of 100 millisecond inter-spike interval (ISI). Network bursts were defined as a minimum of 10 spikes with a maximum of 100 milliseconds ISI and at least 35% of electrodes in synchrony. Synchronization index was calculated as per Axion Biosystems MEA manual and a previously published formula for calculating spike synchrony (Paiva et al., 2010). Offline processing was performed using Axion Biosystems Neural Metric Tool.

### Lactate dehydrogenase (LDH) assay

Cell media from an MEA experiment plate was collected and LDH activity was detected using an LDH Cytotoxicity Assay Kit (Cayman Chemical) according to the manufacturer’s protocol. The absorbance value of each well was measured at a wavelength of 490 nm using a microplate reader.

### *In vitro* electrophysiology

iNeurons were replated on DIV 4 onto polyornithine/laminin coated coverslips in a 24-well plate at a density of 100,000/well with 0.5 mL of iNI media. On DIV 5, primary mouse astrocytes were added at a density of 50,000/well to support iNeurons’ viability and maturation. Half media changes were performed every other day and wells were maintained until DIV 24–26 for recordings. At DIV 9, iNI was supplemented with 2.5% FBS which was adapted from Zhang et al. (2013). Whole-cell patch-clamp recordings were performed at room temperature using Multiclamp 700B amplifier (Molecular Devices) from borosilicate patch electrodes (P-97 puller and P-1000 puller; Sutter Instruments) containing a potassium-based intracellular solution (in mM): 123 K-gluconate, 10 KCl, 10 HEPES; 1 EGTA, 1 MgCl2, 0.1 CaCl2, 1 Mg-ATP, and 0.2 Na4GTP (pH 7.2). 0.06% sulforhodamine dye was added to the intracellular solution to confirm the selection of multipolar neurons. The extracellular solution consisted of (in mM): 140 NaCl, 5 KCl, 1.25 NaH_2_PO_4_, 1 MgCl_2_, 2 CaCl_2_, 10 glucose, and 10 HEPES (pH 7.4). Data were digitized at 10–20 kHz and low-pass filtered at 1–2 kHz. Recordings were omitted if access resistance was > 30 MΩ. Whole-cell recordings were clamped at −70 mV and corrected for a calculated −10 mV junction potential. Rheobase was determined by a step protocol with 5 pA increments, where the injected current had a 25 ms duration. Action potential waveform parameters were all analyzed in reference to the threshold. Repetitive firing step protocols ranged from −20 pA to +50 pA with 5 pA increments for the isogenic KO line. This was adapted for the patient-derived iNeurons as it took more current to elicit their rheobase. The repetitive firing step protocol ranged from −40 pA to +90 pA with 10 pA increments with current injections lasting 500 ms. No more than two iNeurons per coverslip were used to reduce the variability. Data were analyzed using the Clampfit software (Molecular Devices), while phase-plane plots were generated in the OriginPro software (Origin Lab).

### Statistical analysis

Data are expressed as mean ± SEM with the exception of the CTRL-F1 and R607* ddPCR data which show mean ± standard deviation. Three viral NGN2 transductions were used as biological replicates for statistical analysis. We used the Student’s unpaired *t*-test, two-way ANOVA (repeated measures for when analyzing MEA data and repetitive firing properties), and *post hoc* Sidak tests in GraphPad Prism 8 statistical software for statistical analyses. Sidak was used to correct for multiple comparisons. Grubbs’ test was used to remove outliers. The *p*-values in the figure legends are from the specified tests, and *p* < 0.05 was considered statistically significant.

## Results

### Generation and characterization of isogenic *SCN2A* knockout and ASD patient-derived *de novo* truncating *SCN2A*^+/*R*607*^ iPSCs

We recruited a male ASD proband with a *de novo* truncating variant in *SCN2A* at amino acid position 607 (c.1819C > T, R607X; *SCN2A*^+/*R*607*^) with their sex-matched parental control through the MSSNG project (Yuen et al., 2017). iPSCs were generated as previous described (Deneault et al., 2018), and all iPSCs had normal karyotype, expressed the pluripotency markers (OCT4 and NANOG), and were mycoplasma free (Supplementary Figures 1A–C). We confirmed that the iPSCs from the proband carried the *SCN2A* variant (Supplementary Figure 1D). In addition to the patient-derived and parental control iPSCs, we generated a *SCN2A* knockout (KO) iPSC line (*SCN2A*^–/–^) using CRISPR/Cas9 to insert a STOP-tag into exon 5 into a previously used neurotypical healthy control iPSC line (named 50B) (Deneault et al., 2018), which was confirmed through Sanger sequencing (Supplementary Figure 1D). Nomenclature used in this study can be found in Figure 1C.

### Reduced *SCN2A* expression disrupts human iNeuron synaptic morphology

**FIGURE 2:**
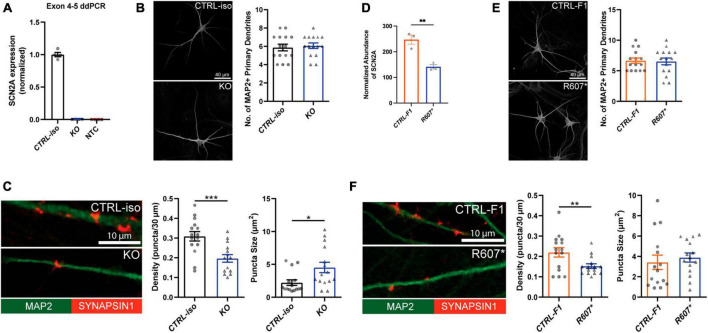
Loss of SCN2A by CRISPR knockout or a patient R607* *de novo* truncating variant in human iPSC-iNeurons impair synaptic morphology. **(A)** Digital droplet PCR quantification of exon 4–5 of the isogenic KO line showing normalized *SCN2A* expression. CTRL-iso (*n* = 4) and KO (*n* = 4) iNeuron cultures. NTC = no template control. **(B)** Representative image of immunocytochemistry for dendrite complexity and analysis for MAP2-positive primary dendrites [*t*_(28)_ = 0.4243, *p* = 0.6746]. CTRL-iso (*n* = 15) and KO (*n* = 15) iNeurons, 3 viral transductions. Scale bar, 40 μm. **(C)** Representative images and quantification of synaptic puncta density [*t*_(28)_ = 3.720, *p* = 0.0009] and size [*t*_(28)_ = 2.559, *p* = 0.0162] of CTRL-iso (*n* = 15) and KO (*n* = 15) iNeurons, 3 viral transductions. Scale bar, 10 μm. **(D)** Normalized protein abundance from shotgun proteomics quantification of SCN2A in CTRL-F1 (*n* = 3) and R607* (*n* = 3) iNeurons. Data are from 3 independent viral transductions. **(E)** Representative image of immunocytochemistry for dendrite complexity and analysis for MAP2-positive primary dendrites [*t*_(28)_ = 0.1903, *p* = 0.8504]. CTRL-F1 (*n* = 15) and R607* (*n* = 15) Neurons, 3 viral transductions. Scale bar, 40 μm. **(F)** Representative images and quantification of synaptic puncta density [*t*_(28)_ = 2.609, *p* = 0.0144] and size of CTRL-F1 (*n* = 15) and R607* (*n* = 15) iNeurons, 3 viral transductions. Scale bar, 10 μm.

We used a modified Neurogenin2 (NGN2) induction protocol (Zhang et al., 2013) to generate iNeurons. We validated *SCN2A* expression in iNeurons using droplet digital PCR (ddPCR) due to difficulty in performing reliable western blots for SCN2A, a large (> 200 kDa) membrane bound protein. Using a probe targeting exon 4–5, we found very little transcript detected in our isogenic KO compared to its control, demonstrating our 3x STOP-tag knock-in was effective at disrupting *SCN2A* expression (Figure 2A). Using patient R607* iNeurons, we performed whole cell shotgun proteomics, and found expression of SCN2A to be reduced by ∼50% compared to control iNeurons (Figure 2D).

Next, we examined dendrite growth and synapse formation since previous studies have shown that *Scn2a*^±^ animal studies have immature spine development in cortical excitatory neurons (Spratt et al., 2019). We used DIV 26–28 iNeurons and identified synapses by staining for the presynaptic terminal marker Synapsin1, together with MAP2, a microtubule-associated protein to outline dendrites. We did not observe significant differences in primary dendrite formation for KO or R607* iNeurons compared to their respective controls (Figures 2B, E). However, the isogenic KO and patient R607* iNeurons displayed decreased Synapsin1-positive synaptic density, with fewer presynaptic terminals being formed (Figures 2C, F). The size of presynaptic terminals was increased in KO iNeurons, but there was no change in patient R607* iNeurons (Figures 2C, F and Supplementary Figure 4A). These results suggest a potential synaptic functional deficit in iNeurons lacking *SCN2A*, with a stronger effect present in homozygous KO iNeurons.

### Loss of *SCN2A* impairs normal neuronal activity and action potential dynamics

**FIGURE 3:**
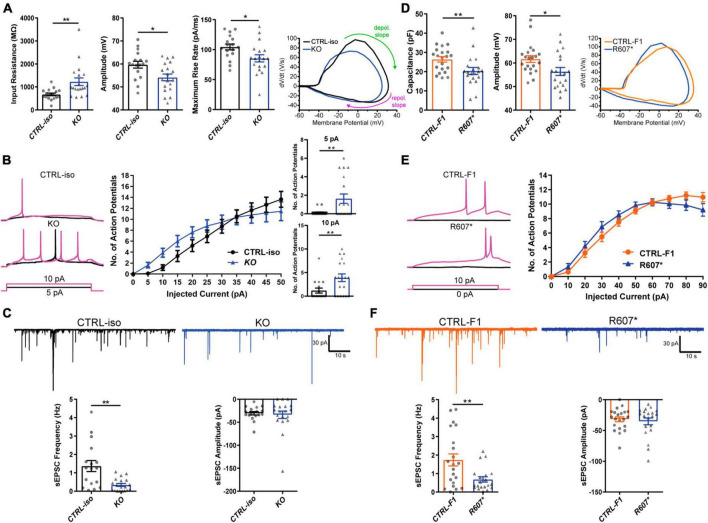
Functional loss of SCN2A disrupts neuronal activity and synaptic transmission. **(A)** Membrane and action potential properties of CTRL-iso and SCN2A KO iNeurons (*n* = 17 and 19, respectively), 3 viral transductions. **Left:** bar graph of recorded input resistance [*t*_(34)_ = 3.019, *p* = 0.0048]. **Middle:** bar graph of measured action potential amplitude [*t*_(34)_ = 2.667, *p* = 0.0116]. **Right:** bar graph of the maximum rise rate [*t*_(34)_ = 2,430, *p* = 0.0205] and the associated phase plane plot of action potential kinetics. Labeled on the phase plane plot are the depolarization (depol.) and repolarization (repol.) slopes in the green and purples arrows, respectively. Data represent means ± SEM. *p < 0.05, **p < 0.01, Student’s *t*-test. **(B)** Repetitive firing properties of SCN2A KO iNeurons. Data represent means ± SEM. *p < 0.05, **p < 0.01, two-way repeated measures ANOVA with *post hoc* Sidak correction [*F*(1, 39) = 0.0512, *p* = 0.8222 for effect of genotype; *F*(10, 390) = 3.075, *p* = 0.0009 for interaction of current injection and genotype, significant current injections are shown as bar plots]. **(C)** Synaptic transmission representative traces and analysis. **Left:** sEPSC frequency of synaptic transmission [*t*_(34)_ = 3.520, *p* = 0.0013]. **Right:** sEPSC amplitude of synaptic transmission [*t*_(34)_ = 0.3602, *p* = 0.7209]. CTRL-iso (*n* = 17) and KO (*n* = 19) iNeurons. Data represent means ± SEM. *p < 0.05, **p < 0.01, Student’s *t*-test. **(D)** Membrane and action potential properties of CTRL-F1 and R607* iNeurons (*n* = 21 and 20, respectively). **Left:** bar graph of recorded capacitance [*t*_(39)_ = 2.626, *p* = 0.0123]. **Middle:** bar graph of measured action potential amplitude [*t*_(39)_ = 2.435, *p* = 0.0195]. **Right:** phase plane plot of action potential kinetics. **(E)** Repetitive firing properties of CTRL-F1 and R607* iNeurons. CTRL-F1 (*n* = 21) and R607* (*n* = 20) iNeurons, 3 viral transductions. Data represent means ± SEM. *p < 0.05, two-way repeated measures ANOVA with *post hoc* Sidak correction [*F*(1, 34) = 0.2956, *p* = 0.5902 for effect of genotype; *F*(10, 340) = 2.525, *p* = 0.0061 for interaction of current injection and genotype]. **(F)** Synaptic transmission representative traces and analysis. **Left:** sEPSC frequency of synaptic transmission [*t*_(39)_ = 2.930, *p* = 0.0056]. **Right:** sEPSC amplitude of synaptic transmission [*t*_(39)_ = 0.5059, *p* = 0.6158]. CTRL-F1 (*n* = 21) and R607* (*n* = 20) iNeurons, 3 viral transductions. Data represent means ± SEM. *p < 0.05, **p < 0.01, Student’s *t*-test.

We next examined the biophysical properties of isogenic KO- and patient-derived R607* iNeurons. Whole-cell patch-clamp recordings were done at DIV 24–28, with all iNeurons being co-cultured with mouse glia to promote maturation and synapse formation. We measured intrinsic membrane properties, including active and passive electrophysiological properties. KO iNeurons expressed higher input resistances compared to their isogenic control (Figure 3A), suggesting that KO iNeurons require less current input to elicit the same response as CTRL-iso iNeurons. We also found KO iNeurons had decreased action potential amplitudes (Figure 3A), however, other intrinsic properties remained unchanged (Supplementary Figures 2A–D). We analyzed action potential characteristics and found KO iNeurons had reduced maximum rise rates, (Figure 3A) which was similar to previous mouse studies (Spratt et al., 2021). There was no difference in the maximum decay rates or the half-width, suggesting that sodium channels were solely impaired (Supplementary Figures 2E, F). To depict changes of action potential waveforms, we used phase-plane plots generated from the first derivative of the membrane potential (dV/dt) versus the membrane potential. This visualization depicts decreases in peak amplitude and the maximum value of dV/dt (Figure 3A). Next, we examined repetitive firing using a step protocol and found increases in the maximum number action potentials fired at 5 pA and 10 pA current injection steps for KO iNeurons (Figure 3B). These results suggest an intrinsic hyperexcitability phenotype. While this is paradoxical given that sodium channels are required for action potential generation; however, this phenomenon is observed in conditional *Scn2a*^–/–^ mice (Spratt et al., 2021; Zhang et al., 2021).

We next examined patient-derived iNeurons expressing the *de novo* R607* truncating variant and the respective familial control. Passive and active membrane properties were examined, and we determined the capacitance of R607* iNeurons was decreased relative to CTRL-F1 iNeurons (Figure 3D). This suggests that R607* iNeurons are smaller, but other membrane properties remained unchanged (Supplementary Figures 2G–I). When we measured action potential characteristics, we found a decrease in action potential amplitude in R607* iNeurons (Figure 3D), similar to isogenic KO iNeurons. However, maximum rise rate (Figure 3D) and other action potential characteristics remained unchanged in patient R607* iNeurons (Supplementary Figures 2J–M), which distinguished this line from the KO. Measurement of the repetitive firing capabilities of R607* iNeurons revealed no statistical differences (Figure 3E), whereas there was a difference in isogenic KO iNeurons (Figure 3B), suggesting a milder phenotype in regard to excitability.

### Early truncation of SCN2A impairs excitatory synaptic transmission

We recorded spontaneous excitatory postsynaptic currents (sEPSCs) as a proxy for synaptic transmission to understand the impact caused by the R607* variant. Isogenic KO iNeurons displayed a reduction in the frequency but not the amplitude of synaptic events (Figure 3C), similar to KO iNeurons derived from a different genetic background that we previously reported (Deneault et al., 2018). Recordings of R607* iNeurons revealed a similar decrease in sEPSC frequency with no change in amplitude (Figure 3F). These results reveal that the KO and the R607* variant is largely loss-of-function and causes a reduction in synaptic connectivity and transmission.

### Severe loss of SCN2A impairs spontaneous neuronal network activity in iNeurons

**FIGURE 4:**
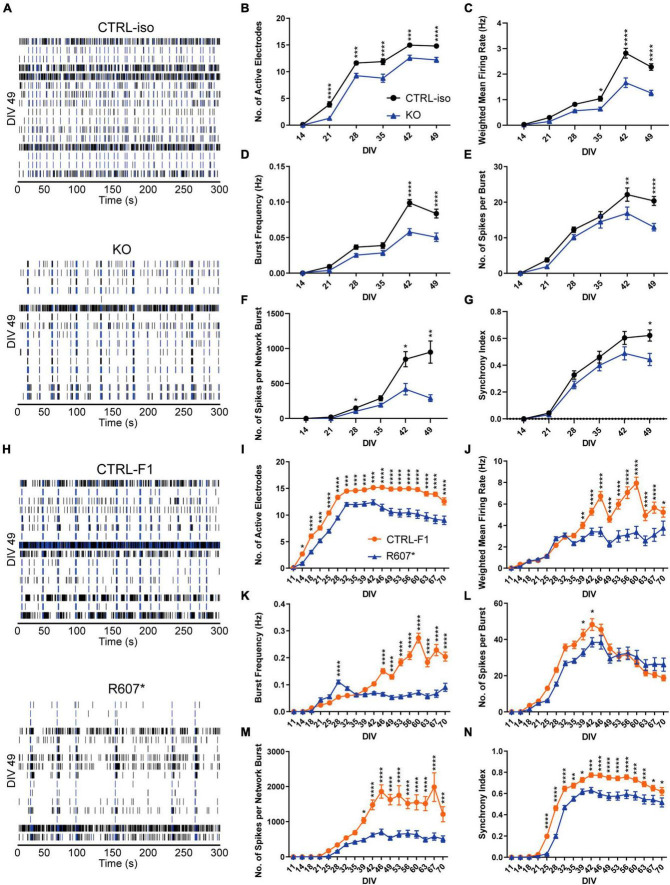
Complete and partial loss of SCN2A impairs the development of spontaneous network activity via multi-electrode array. **(A)** Example raster plots of recordings of neuronal network activity at DIV 49 for CTRL-iso and SCN2A KO iNeurons. **(B–G)** Quantification of MEA parameters for CTRL-iso and KO iNeurons: **(B)** number of active electrodes [*F*(1, 83) = 35.55, *p* < 0.0001 for effect of genotype; *F*(5, 415) = 4.778, *p* = 0.0003 for interaction of time and genotype], **(C)** weighted mean firing rate [*F*(1, 83) = 37.31, *p* < 0.0001 for effect of genotype; *F*(5, 415) = 14.32, *p* < 0.0001 for interaction of time and genotype], **(D)** burst frequency [*F*(1, 83) = 27.78, *p* < 0.0001 for effect of genotype; *F*(5, 415) = 12.68, *p* < 0.0001 for interaction of time and genotype], **(E)** number of spikes per burst [*F*(1, 83) = 6.570, *p* = 0.0122 for effect of genotype; *F*(5, 415) = 5.011, *p* = 0.0002 for interaction of time and genotype], **(F)** number of spikes per network burst [*F*(1, 83) = 14.55, *p* = 0.0003 for effect of genotype; *F*(5, 415) = 11.85, *p* < 0.0001 for interaction of time and genotype], **(G)** synchrony index [*F*(1, 83) = 3.667, *p* = 0.0586 for effect of genotype; *F*(5, 415) = 4.170, *p* = 0.0010 for interaction of time and genotype]. CTRL-iso (*n* = 47 wells) and KO (*n* = 43 wells) iNeurons, 3 viral transductions. Data represent means ± SEM. *p < 0.05, **p < 0.01, ***p < 0.001, ****p < 0.001, two-way repeated measures ANOVA with *post hoc* Sidak correction. **(H)** Example raster plots of recordings of neuronal network activity at DIV 49 for CTRL-F1 and R607* iNeurons. **(I–N)** Quantification of MEA parameters for CTRL-F1 and R607* neurons: **(I)** number of active electrodes [*F*(2, 62) = 24.71, *p* < 0.0001 for effect of genotype; *F*(34, 1,054) = 18.50 = 4.778, *p* < 0.0001 for interaction of time and genotype], **(J)** weighted mean firing rate [*F*(1, 125) = 21.49, *p* < 0.0001 for effect of genotype; *F*(17, 2125) = 16.10, *p* < 0.0001 for interaction of time and genotype], **(K)** burst frequency [*F*(2, 62) = 8.794, *p* = 0.0004 for effect of genotype; *F*(34, 1,054) = 8.359, *p* < 0.0001 for interaction of time and genotype], **(L)** number of spikes per burst [*F*(2, 62) = 23.98, *p* < 0.0001 for effect of genotype; *F*(34, 1,054) = 17.17, *p* < 0.0001 for interaction of time and genotype], **(M)** number of spikes per network burst [*F*(2, 62) = 25.04, *p* < 0.0001 for effect of genotype; *F*(34, 1,054) = 15.83, *p* < 0.0001 for interaction of time and genotype], **(N)** synchrony index [*F*(2, 62) = 21.68, *p* < 0.0001 for effect of genotype; *F*(34, 1,054) = 18.94, *p* < 0.0001 for interaction of time and genotype]. CTRL-F1 (*n* = 48 wells) and R607* (*n* = 46 wells) iNeurons, 3 viral transductions. Data represent means ± SEM. *p < 0.05, **p < 0.01, ***p < 0.001, ****p < 0.001, two-way repeated measures ANOVA with *post hoc* Sidak correction.

To better understanding how disruption of *SCN2A* can affect the longitudinal development of neuronal circuits in a network, we used microelectrode arrays (MEAs) using the same plating protocols. Isogenic KO and R607* iNeurons raster plots were taken from DIV 49 depicting network activity within a well, highlighting the activity differences versus the respective controls, and similarities and differences between the genotypes (Figures 4A, H).

Overall, both SCN2A KO and R607* iNeurons displayed similar trends in various parameters. Both displayed a decrease in active electrodes across development (Figures 4B, I), which was not due to reduced neuron survival (Supplementary Figure 5A). Examining the weighted mean firing rate (wMFR), which accounts for the changes in active electrodes, revealed a reduction in KO and R607* iNeurons that began early and persisted (Figures 4C, J) throughout development. This indicates that the loss of *SCN2A* hinders the abundance of neuronal spikes within the culture. We extended the experiment time of patient neurons up to 10-weeks to determine if longer-term culture conditions alter their developmental trajectory. We found that up to 10-weeks, there was still an overall decrease R607* iNeuron firing, with some recovery in activity near the end (Figures 4I–N).

We further investigated whether network bursting was disrupted since this parameter is indicative of population neuronal activity, and it is disrupted in other models of *SCN2A* deficiency (Deneault et al., 2018; Lu et al., 2019). Additionally, recent investigations into risk genes contributing to ASD and intellectual disability clinical features have showed changes in neuronal network activity, specifically enhancements of bursts (Frega et al., 2020). We examined burst frequency and the number of spikes within each burst since sodium channels are important in action potentials and spike generation. Bursts were defined by having 5 spikes with a maximum of 100 millisecond inter-spike intervals. KO iNeurons displayed a decrease in bursting and the number of spikes per burst (Figures 4D, E). When examining the bursting of patient R607* iNeurons, unlike the KO iNeurons, R607* iNeurons exhibited a small increase in bursting early in development (Figure 4K), which dissipated and became reduced compared to CTRL-F1 iNeurons. Furthermore, R607* iNeurons displayed a decrease in the number of spikes per burst for a transient window between DIV 39–46 (Figures 4E vs. L). Secondary burst parameters were also examined and showed similar bursting patterns (Supplementary Figures 3A, B, E, F).

Network bursting and synchronization occurs in later stages of neuronal development, where this is crucial for the organization and regulation of excitation (Stegenga et al., 2008; Masquelier and Deco, 2013; Kirwan et al., 2015; Kim et al., 2019). Network bursts were defined as having 4 or more electrodes to burst within 100 ms of one another. We found that the number of spikes per network burst was reduced in KO and R607* iNeurons at approximately 4 weeks (Figures 4F, M). Network burst frequency and network burst duration were also recorded and corroborated impaired network communication (Supplementary Figures 3C, D, G, H), similar to our previously reported isogenic KO iNeurons in a different genetic background (Deneault et al., 2018).

Lastly, we examined synchronization, which is a parameter defined as the probability for neighboring electrodes to detect activity in quick succession based on prior electrode activity. This is measured as an index ranging from 0 to 1, where 1 represents neighboring electrodes detecting activity based on a prior active electrode, 100% of the time. Interestingly, we found that all iNeurons displayed deviation from their respected control lines, suggesting that synchronization of neurons in the network was impaired by any type of SCN2A disruption (Figures 4G, N).

## Discussion

In this study, we present data from two genetic human cellular models of *SCN2A*, to understand the channelopathy contributions to neurodevelopmental disorders. Previous models suggest that *SCN2A* variants that cause an enhancement of neuronal excitability result in epilepsy and are gain-of-function, whereas, ASD-linked variants are loss-of-function and reduce channel activity (Ben-Shalom et al., 2017; Deneault et al., 2018; Ogiwara et al., 2018; Rubinstein et al., 2018; Sanders et al., 2018; Begemann et al., 2019; Lu et al., 2019; Spratt et al., 2019, 2021; Wang et al., 2021; Zhang et al., 2021). This classification does not fully characterize the channelopathy, especially where an estimated 20–30% of ASD/ID patients develop late-onset seizures in addition to SCN2A deficiency (Wolff et al., 2017; Sanders et al., 2018; Zhang et al., 2021). Our data indicates that patient iNeurons harboring an early truncating (R607*) ASD-linked *SCN2A* variant display reduced channel function and impair action potential characteristics, synaptic dysfunction and neuronal signaling networks similar to a homozygous KO of *SCN2A*. The R607* mutation produces a truncated protein with only the first of four domains which likely results in a non-function protein. This mutation removes critical regions such as the Ankyrin G-binding motif that is essential for localization to the axon initial segment (Bender and Trussell, 2012). Similarly, Lu et al. (2019), generated glutamatergic iNs from two human embryonic stem cell lines heterozygous for *SCN2A* and reported a reduction in the firing ability of those neurons. In this study, the truncating mutations were introduced at amino acid positions at 877 and 1,077, suggesting that removal of nearly 50% of the protein structure results in impair neuronal activity. Put together, these findings support the hypothesis that an early LoF variant in *SCN2A* impairs neuronal communication through reduced channel activity, however, the extent to which an LoF variant along the protein structure can affect the channel function remains unknown.

Human iPSC-derived KO iNeurons displayed a paradoxical intrinsic hyperexcitability phenotype, similar to mouse *Scn2a*^–/–^ neurons (Spratt et al., 2021; Zhang et al., 2021), but not in patient iNeurons as they have a heterozygous loss of *SCN2A* function. It has been reported that NGN2-derived glutamatergic neurons around 2–3 weeks *in vitro* resemble second trimester fetal neurons (Hulme et al., 2022). Our MEA results suggest that this may be a vulnerable time point in fetal neurodevelopment that may extend to other *SCN2A*-associated ASD cases which some *SCN2A*-mediated disorders report seizure-like activity *in utero* (Sanders et al., 2018). Further characterization using MEA network analysis of other ASD-associated truncating variants in *SCN2A* using iNeurons are needed to support this hypothesis.

Patient-derived R607* iNeurons had a decrease in synaptic transmission, but it is unknown if this is a direct or indirect impact. Extrapolating and comparing to mouse *Scn2a* haploinsufficiency, the reduction in excitatory synaptic transmission in human R607* iNeurons could be driven by changes in the AMPA:NMDA ratio, inferring an abundance of silent synapses due to an abundance of AMPA-lacking spines (Kerchner and Nicoll, 2008; Hanse et al., 2013; Spratt et al., 2019). We noted both KO and R607* iNeurons generated less spontaneous electrical activity, which could suggest SCN2A deficiency, without seizures, dampens neural networks and produces small quiescent pockets within the network (Deneault et al., 2018). Further, it is possible that SCN2A regulates neuronal activity-dependent mechanisms such as activity-dependent transcription. Given the inference of silent synapse above, it is possible that reduced neuronal activity through loss or reduced SCN2A channel activity drives activity-dependent changes that alter AMPA or NMDA receptor expression. Future studies should examine how other *SCN2A* missense variants impact human neuron synaptic function, as these variants do not cause protein truncation but lead to effects on channel function (Ben-Shalom et al., 2017; Echevarria-Cooper et al., 2022). Additionally, future studies should investigate the transcriptomic and proteomic changes that occur in an activity-dependent manner in *SCN2A*-deficient neurons to identify possible changes in expression programs that may regulate cellular dysfunction in these neurons.

The NGN2 model system has both advantages and disadvantages for modeling *SCN2A* variants. The system is robust in producing functional neurons from iPSCs, and iNeurons function well for in multielectrode array and patch-clamp experiments. However, the limitations are that iNeurons are more heterogeneous than previously thought, and do not resemble a specific neuronal sub-type (Lin et al., 2021). Further, their transcriptional signatures can be shared with sensory neurons (Lin et al., 2021), and linked to NGN2 expression levels. Further, a small portion of iNeurons can have two or more axons (Rhee et al., 2019). To address these limitations, the use of a more robust physiologically relevant *in vitro* system such as mixed co-culture of induced glutamatergic and GABAergic neurons (Wang et al., 2022) would provide deeper insights into the disease state.

In summary, we have shown functional similarities in iNeurons that completely lack *SCN2A* and an ASD-associated R607* truncating variant at both the single neuron and population level. Neurons deficient for *SCN2A* exhibit an electrophysiological immature phenotype compared to their controls. Whether this immature phenotype exists only at the functional level or persists throughout development and impacts brain development remains to be discovered. Further analysis of different *SCN2A* variants in neurons will be required to understand the impact on neuron development to better predict the potential effects on circuit dysfunction in the brain.

## Data availability statement

The raw data supporting the conclusions of this article will be made available by the authors, without undue reservation.

## Ethics statement

The studies involving humans were approved by the University Health Network Research Ethics Board. The studies were conducted in accordance with the local legislation and institutional requirements. The human samples used in this study were acquired from primarily isolated as part of your previous study for which ethical approval was obtained. Written informed consent for participation was not required from the participants or the participants’ legal guardians/next of kin in accordance with the national legislation and institutional requirements.

## Author contributions

CB performed all electrophysiology experiments and data analysis. CB and JU performed morphological experiments and data analysis. NM, ER, AA, SK, and AC contributed to experiments. BD and SW performed CRISPR-editing for the 50B line. SS provided patient access and samples. CB, JU, and KS wrote the manuscript. All authors contributed to the article and approved the submitted version.

## Footnotes

1
www.mss.ng

## Supplementary material

Supplementary Figure 1 iPSC validation of *SCN2A* deficient genetic models. **(A)** **Left:** Representative immunocytochemistry of *SCN2A* iPSC lines for OCT4, NANOG and DAPI. **Right:** Chromosomal images from G-banding karyotyping. Scale bar, 100 μm. **(B)** Mycoplasma validation of *SCN2A* iPSC lines. **(C)** Sanger sequencing validation and quantification of heterozygosity of the R607* variant iPSCs. **(D)** Sanger sequencing validation of the STOP-tag in the isogenic *SCN2A* KO iPSCs.

Supplementary Figure 2 Intrinsic properties of KO and R607* iNeurons. **(A–F)** Patch-clamp electrophysiology of CTRL-iso and KO iNeurons (*n* = 17 and 19, respectively), 3 viral transductions: **(A)** Capacitance of iNeurons [*t*_(34)_ = 0.6646, *p* = 0.5108, **(B)** resting membrane potential [*t*_(34)_ = 0.3908, *p* = 0.6984], **(C)** rheobase [*t*_(34)_ = 1.131, *p* = 0.2658, **(D)** action potential threshold [*t*_(34)_ = 0.8961, *p* = 0.3765], **(E)** action potential half-width [*t*_(34)_ = 0.9960, *p* = 0.3263], **(F)** maximum decay rate [*t*_(34)_ = 1.301, *p* = 0.2019]. Data represent means ± SEM. Student’s *t*-test. **(G–M)** Patch-clamp electrophysiology of CTRL-F1 and R607* iNeurons (*n* = 21 and *n* = 20, respectively), 3 viral transductions: **(G)** iNeuron input resistance [*t*_(39)_ = 1.930, *p* = 0.0609], **(H)** resting membrane potential [*t*_(39)_ = 0.3849, *p* = 0.7024], **(I)** rheobase [*t*_(39)_ = 0.1304, *p* = 0.8969], **(J)** threshold of action potential [*t*_(39)_ = 0.7773, *p* = 0.4416], **(K)** half-width of action potential [*t*_(39)_ = 0.1406, *p* = 0.8889], **(L)** maximum rise rate [*t*_(39)_ = 1.497, *p* = 0.1424], **(M)** maximum decay rate [*t*_(39)_ = 1.385, *p* = 0.1741]. Data represent means ± SEM. **p* < 0.05, Student’s *t*-test.

Supplementary Figure 3 Spontaneous network activity of *SCN2A* deficiency via multi-electrode array. **(A–D)** Analysis of additional isogenic KO iNeuron spontaneous network parameters. CTRL-iso (*n* = 47 wells) and KO (*n* = 43 wells), 3 viral transductions: **(A)** Burst duration [*F*(1, 84) = 15.03, *p* = 0.0002 for effect of genotype; *F*(5, 420) = 1.411, *p* = 0.2191 for interaction of time and genotype], **(B)** Inter-burst interval [*F*(1.83) = 0.5027, *p* = 0.4803 for effect of genotype; *F*(5, 415) = 2.349, *p* = 0.0403 for interaction of time and genotype], **(C)** Network burst frequency [*F*(1, 83) = 8.759, *p* = 0.0040 for effect of genotype; *F*(5, 415) = 2.033, *p* = 0.0713 for interaction of time and genotype], **(D)** Network burst duration [*F*(1, 83) = 10.10, *p* = 0.0021 for effect of genotype; *F*(5, 415) = 4.783, *p* = 0.0003 for interaction of time and genotype]. **(E–H)** Analysis of additional R607* iNeuron spontaneous network parameters. CTRL-F1 (*n* = 48 wells) and R607* (*n* = 46 wells), 3 viral transductions: **(E)** Burst duration [*F*(2, 62) = 29.56, *p* = 0.0016 for effect of genotype; *F*(34, 1,054) = 15.79, *p* < 0.0001 for interaction of time and genotype], **(F)** Inter-burst interval [*F*(2, 62) = 0.9033, *p* = 0.4105 for effect of genotype; *F*(34, 1,054) = 2.599, *p* < 0.0001 for interaction of time and genotype], **(G)** Network burst frequency [*F*(2, 62) = 6.409, *p* = 0.0030 for effect of genotype; *F*(34, 1,054) = 6.440, *p* < 0.0001 for interaction of time and genotype], **(H)** Network burst duration [*F*(2, 62) = 7.128, *p* = 0.0016 for effect of genotype; *F*(34, 1,054) = 4.988, *p* < 0.0001 for interaction of time and genotype]. Data represent means ± SEM. **p* < 0.05, ***p* < 0.01, ****p* < 0.001, *****p* < 0.001, two-way repeated measures ANOVA with *post hoc* Sidak correction.

Supplementary Figure 4 Synaptic morphology of KO iNeurons. Related to Figure 2C. **(A)** Representative image of synaptic puncta size of CTRL-iso and KO neurons.

Supplementary Figure 5 No difference in cell death measured by the LDH assay in R607* MEAs. Related to Figure 4. **(A)** Quantification of LDH absorption between CTRL-F1 and R607* neurons at week 70 in MEA experiments. CTRL-F1 (*n* = 22 wells) and R607* (*n* = 21 wells), 1 viral transduction. Data represent means ± SEM. Student’s *t*-test.
