# Supplementary figures and images for: Disruption of the autism-associated gene SCN2A alters synaptic development and neuronal signaling in patient iPSC-glutamatergic neurons
Source: Front Cell Neurosci. 2024 Jan 16;17:1239069. doi: 10.3389/fncel.2023.1239069 (PMC10824931; doi:10.3389/fncel.2023.1239069)

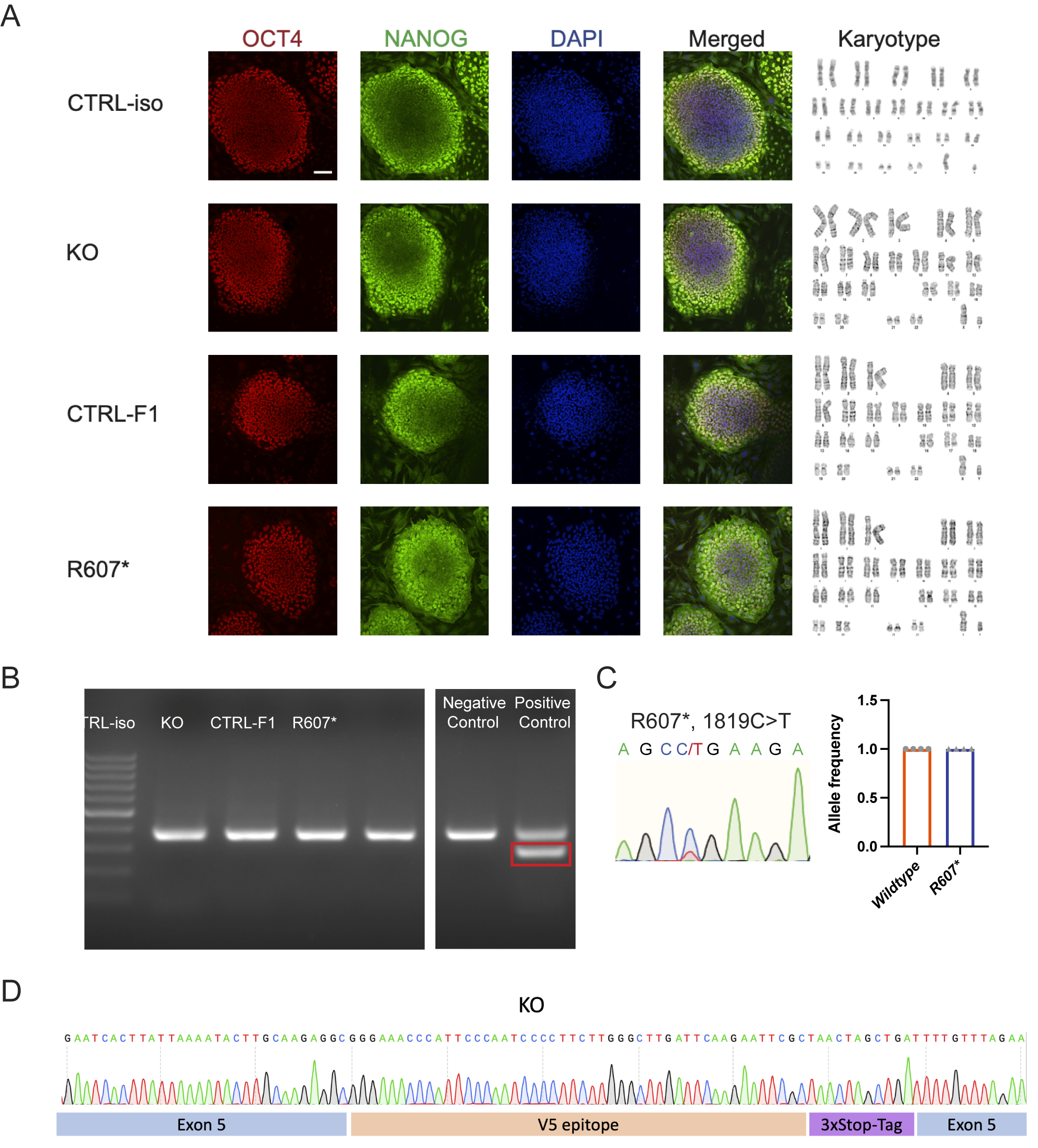

Supplement: Supplementary Figure 1 — iPSC validation of SCN2A deficient genetic models. (A) Left: Representative immunocytochemistry of SCN2A iPSC lines for OCT4, NANOG and DAPI. Right: Chromosomal images from G-banding karyotyping. Scale bar, 100 μm. (B) Mycoplasma validation of SCN2A iPSC lines. (C) Sanger sequencing validation and quantification of heterozygosity of the R607* variant iPSCs. (D) Sanger sequencing validation of the STOP-tag in the isogenic SCN2A KO iPSCs. [file Image_1.TIF]

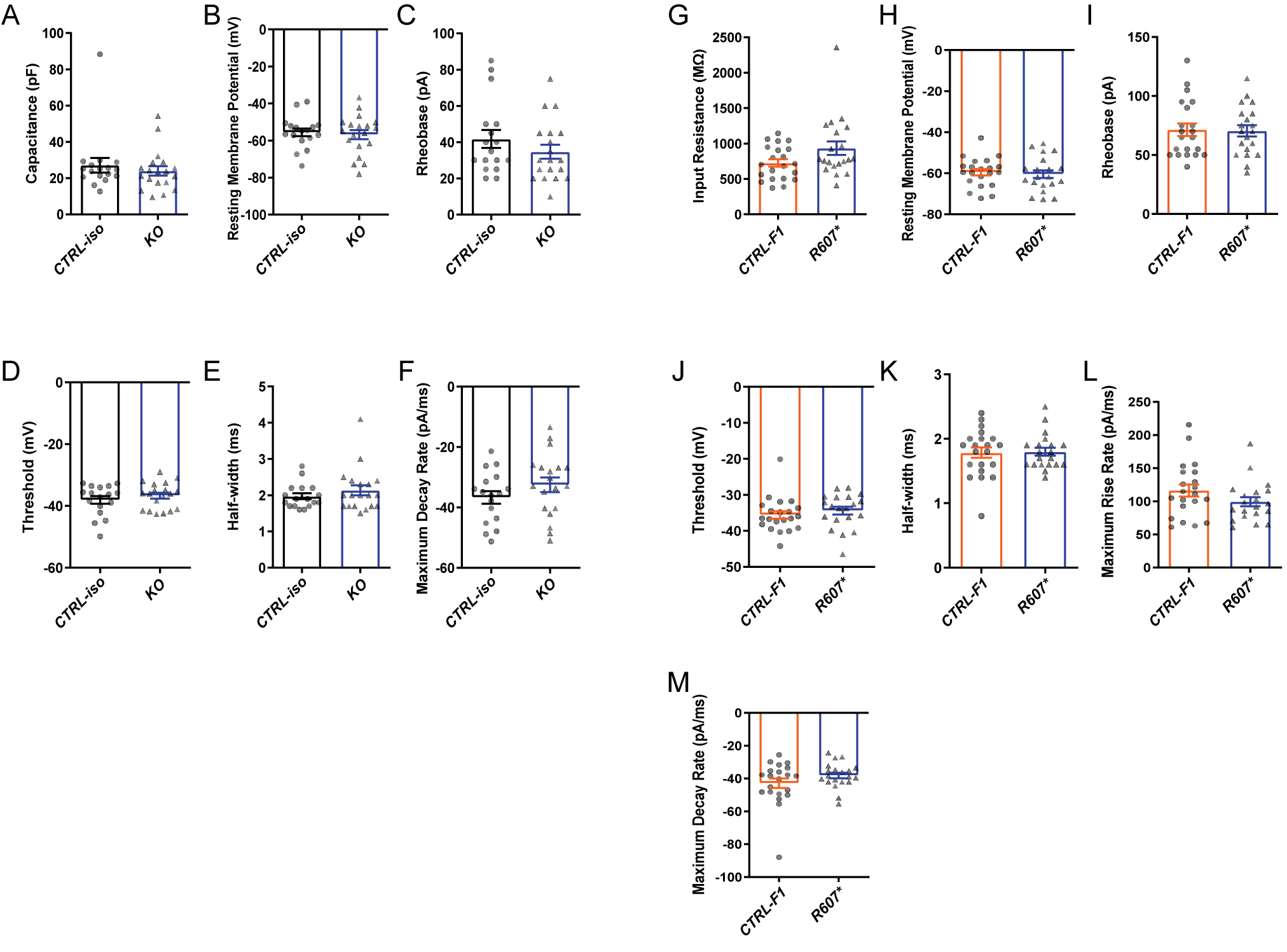

Supplement: Supplementary Figure 2 — Intrinsic properties of KO and R607* iNeurons. (A–F) Patch-clamp electrophysiology of CTRL-iso and KO iNeurons (n = 17 and 19, respectively), 3 viral transductions: (A) Capacitance of iNeurons [t(34) = 0.6646, p = 0.5108, (B) resting membrane potential [t(34) = 0.3908, p = 0.6984], (C) rheobase [t(34) = 1.131, p = 0.2658, (D) action potential threshold [t(34) = 0.8961, p = 0.3765], (E) action potential half-width [t(34) = 0.9960, p = 0.3263], (F) maximum decay rate [t(34) = 1.301, p = 0.2019]. Data represent means ± SEM. Student’s t-test. (G–M) Patch-clamp electrophysiology of CTRL-F1 and R607* iNeurons (n = 21 and n = 20, respectively), 3 viral transductions: (G) iNeuron input resistance [t(39) = 1.930, p = 0.0609], (H) resting membrane potential [t(39) = 0.3849, p = 0.7024], (I) rheobase [t(39) = 0.1304, p = 0.8969], (J) threshold of action potential [t(39) = 0.7773, p = 0.4416], (K) half-width of action potential [t(39) = 0.1406, p = 0.8889], (L) maximum rise rate [t(39) = 1.497, p = 0.1424], (M) maximum decay rate [t(39) = 1.385, p = 0.1741]. Data represent means ± SEM. *p < 0.05, Student’s t-test. [file Image_2.TIF]

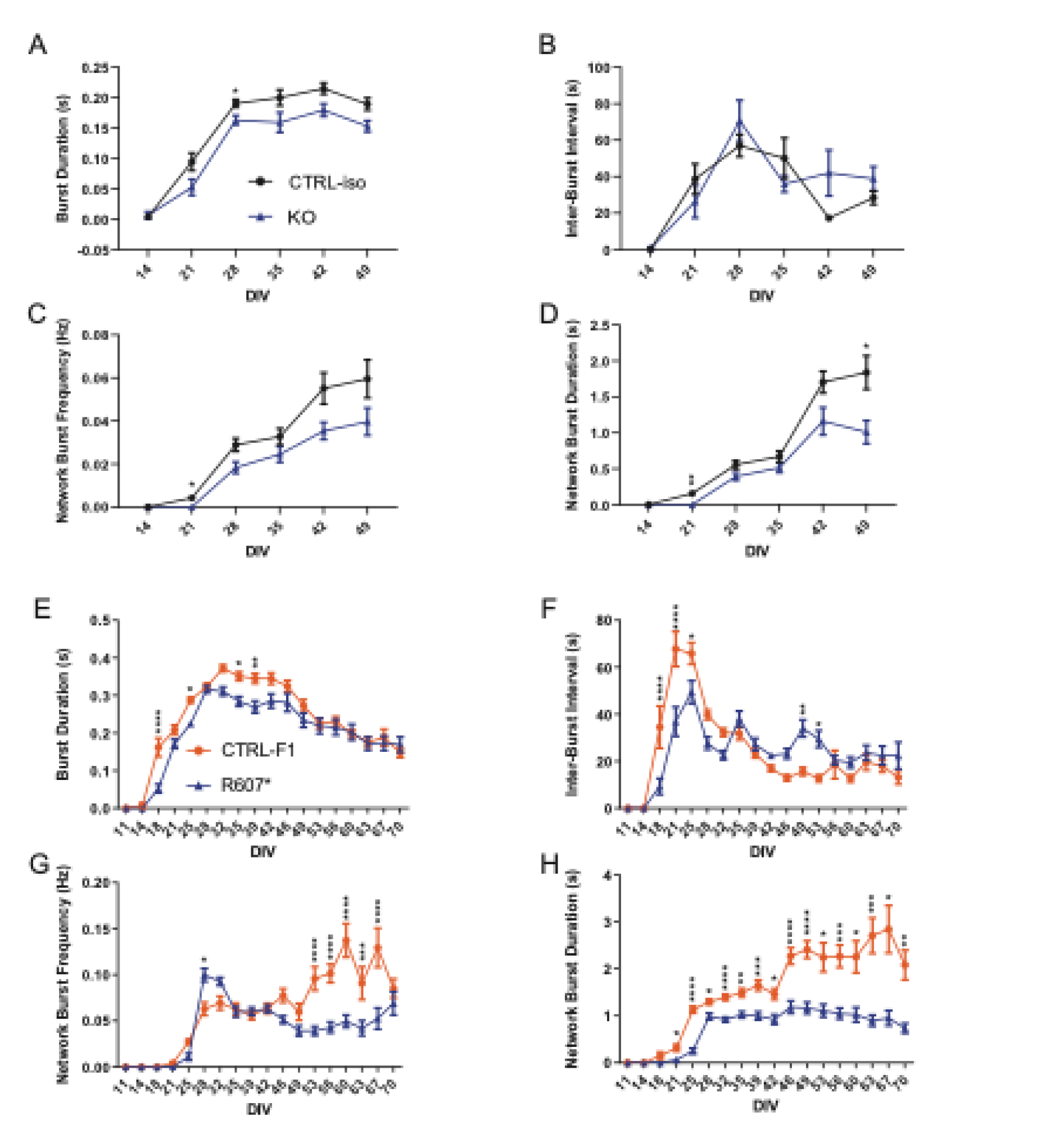

Supplement: Supplementary Figure 3 — Spontaneous network activity of SCN2A deficiency via multi-electrode array. (A–D) Analysis of additional isogenic KO iNeuron spontaneous network parameters. CTRL-iso (n = 47 wells) and KO (n = 43 wells), 3 viral transductions: (A) Burst duration [F(1, 84) = 15.03, p = 0.0002 for effect of genotype; F(5, 420) = 1.411, p = 0.2191 for interaction of time and genotype], (B) Inter-burst interval [F(1.83) = 0.5027, p = 0.4803 for effect of genotype; F(5, 415) = 2.349, p = 0.0403 for interaction of time and genotype], (C) Network burst frequency [F(1, 83) = 8.759, p = 0.0040 for effect of genotype; F(5, 415) = 2.033, p = 0.0713 for interaction of time and genotype], (D) Network burst duration [F(1, 83) = 10.10, p = 0.0021 for effect of genotype; F(5, 415) = 4.783, p = 0.0003 for interaction of time and genotype]. (E–H) Analysis of additional R607* iNeuron spontaneous network parameters. CTRL-F1 (n = 48 wells) and R607* (n = 46 wells), 3 viral transductions: (E) Burst duration [F(2, 62) = 29.56, p = 0.0016 for effect of genotype; F(34, 1,054) = 15.79, p < 0.0001 for interaction of time and genotype], (F) Inter-burst interval [F(2, 62) = 0.9033, p = 0.4105 for effect of genotype; F(34, 1,054) = 2.599, p < 0.0001 for interaction of time and genotype], (G) Network burst frequency [F(2, 62) = 6.409, p = 0.0030 for effect of genotype; F(34, 1,054) = 6.440, p < 0.0001 for interaction of time and genotype], (H) Network burst duration [F(2, 62) = 7.128, p = 0.0016 for effect of genotype; F(34, 1,054) = 4.988, p < 0.0001 for interaction of time and genotype]. Data represent means ± SEM. *p < 0.05, **p < 0.01, ***p < 0.001, ****p < 0.001, two-way repeated measures ANOVA with post hoc Sidak correction. [file Image_3.TIF]

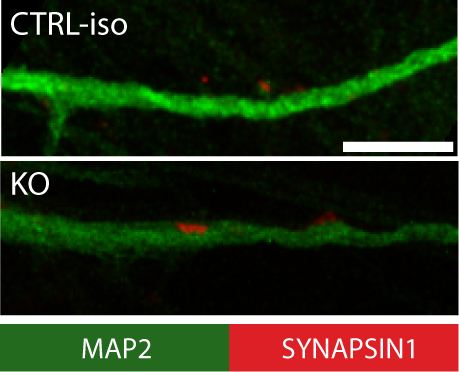

Supplement: Supplementary Figure 4 — Synaptic morphology of KO iNeurons. Related to Figure 2C. (A) Representative image of synaptic puncta size of CTRL-iso and KO neurons. [file Image_4.tiff]

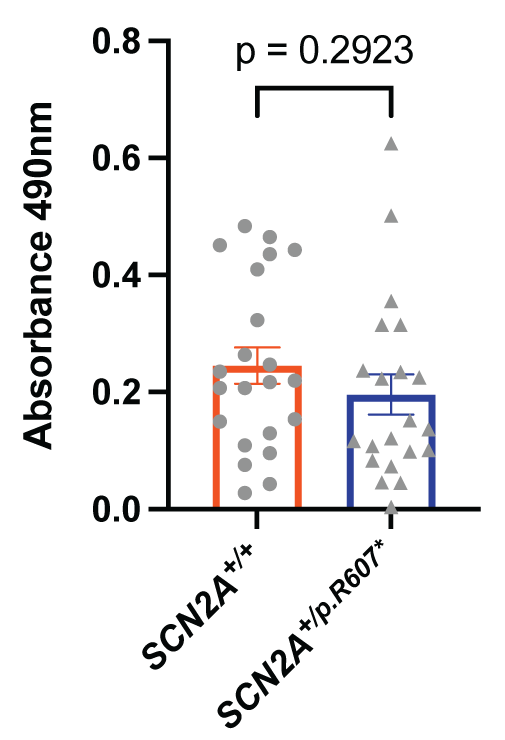

Supplement: Supplementary Figure 5 — No difference in cell death measured by the LDH assay in R607* MEAs. Related to Figure 4. (A) Quantification of LDH absorption between CTRL-F1 and R607* neurons at week 70 in MEA experiments. CTRL-F1 (n = 22 wells) and R607* (n = 21 wells), 1 viral transduction. Data represent means ± SEM. Student’s t-test. [file Image_5.tiff]
